# Supplementary material for: Heparin-based hydrogel scaffolding alters the transcriptomic profile and increases the chemoresistance of MDA-MB-231 triple-negative breast cancer cells
Source: Biomater Sci. 2020 Feb 13;8(10):2786–96. doi: 10.1039/c9bm01481k (PMC7497406; doi:10.1039/c9bm01481k)
Supplement: Supplementary file 2 [file BM-008-C9BM01481K-s002.zip › Supplementary File 4/HP-BhydrogelvControl/Pathways/my_analysis.Gsea.1545201079907/HALLMARK_PROTEIN_SECRETION.html]

Details for gene set HALLMARK\_PROTEIN\_SECRETION[GSEA]

|  || Dataset | expr.class.cls#HYD\_versus\_CONTROL.class.cls#HYD\_versus\_CONTROL\_repos |
| Phenotype | class.cls#HYD\_versus\_CONTROL\_repos |
| Upregulated in class | CONTROL |
| GeneSet | HALLMARK\_PROTEIN\_SECRETION |
| Enrichment Score (ES) | -0.6683001 |
| Normalized Enrichment Score (NES) | -2.8914998 |
| Nominal p-value | 0.0 |
| FDR q-value | 0.0 |
| FWER p-Value | 0.0 |
Table: GSEA Results Summary

  

Fig 1: Enrichment plot: HALLMARK\_PROTEIN\_SECRETION      
 Profile of the Running ES Score & Positions of GeneSet Members on the Rank Ordered List

  

| PROBE | DESCRIPTION (from dataset) | GENE SYMBOL | GENE\_TITLE | RANK IN GENE LIST | RANK METRIC SCORE | RUNNING ES | CORE ENRICHMENT || 1 | ATP6V1B1 | na |  |  | 1205 | 1.738 | -0.0453 | No |
| 2 | STX16 | na |  |  | 2813 | 1.081 | -0.1183 | No |
| 3 | GOSR2 | na |  |  | 4027 | 0.806 | -0.1736 | No |
| 4 | MON2 | na |  |  | 4363 | 0.739 | -0.1836 | No |
| 5 | YKT6 | na |  |  | 5213 | 0.597 | -0.2219 | No |
| 6 | EGFR | na |  |  | 5500 | 0.552 | -0.2312 | No |
| 7 | GBF1 | na |  |  | 5864 | 0.505 | -0.2451 | No |
| 8 | SSPN | na |  |  | 7075 | 0.351 | -0.3048 | No |
| 9 | NAPA | na |  |  | 7192 | 0.340 | -0.3074 | No |
| 10 | CLN5 | na |  |  | 7213 | 0.337 | -0.3050 | No |
| 11 | SGMS1 | na |  |  | 7345 | 0.321 | -0.3086 | No |
| 12 | COPE | na |  |  | 8726 | 0.178 | -0.3790 | No |
| 13 | ARFGEF2 | na |  |  | 9345 | 0.111 | -0.4102 | No |
| 14 | IGF2R | na |  |  | 10025 | 0.038 | -0.4453 | No |
| 15 | ABCA1 | na |  |  | 10121 | 0.026 | -0.4501 | No |
| 16 | AP2M1 | na |  |  | 10716 | -0.031 | -0.4808 | No |
| 17 | COG2 | na |  |  | 10724 | -0.032 | -0.4809 | No |
| 18 | DOPEY1 | na |  |  | 11323 | -0.092 | -0.5112 | No |
| 19 | RER1 | na |  |  | 11337 | -0.094 | -0.5109 | No |
| 20 | KIF1B | na |  |  | 12121 | -0.189 | -0.5500 | No |
| 21 | AP2S1 | na |  |  | 12760 | -0.254 | -0.5808 | No |
| 22 | ANP32E | na |  |  | 12802 | -0.258 | -0.5803 | No |
| 23 | OCRL | na |  |  | 13095 | -0.298 | -0.5925 | No |
| 24 | ICA1 | na |  |  | 13271 | -0.319 | -0.5984 | No |
| 25 | GNAS | na |  |  | 13364 | -0.332 | -0.5998 | No |
| 26 | ARFGEF1 | na |  |  | 13379 | -0.334 | -0.5972 | No |
| 27 | ERGIC3 | na |  |  | 13650 | -0.378 | -0.6074 | No |
| 28 | SEC24D | na |  |  | 14308 | -0.465 | -0.6371 | No |
| 29 | CLTA | na |  |  | 14354 | -0.471 | -0.6346 | No |
| 30 | VAMP4 | na |  |  | 14460 | -0.485 | -0.6351 | No |
| 31 | VPS45 | na |  |  | 14530 | -0.497 | -0.6337 | No |
| 32 | RAB9A | na |  |  | 14531 | -0.497 | -0.6286 | No |
| 33 | STX7 | na |  |  | 14647 | -0.514 | -0.6293 | No |
| 34 | AP3B1 | na |  |  | 14895 | -0.548 | -0.6367 | No |
| 35 | GOLGA4 | na |  |  | 14960 | -0.556 | -0.6343 | No |
| 36 | SCAMP3 | na |  |  | 15102 | -0.580 | -0.6358 | No |
| 37 | CAV2 | na |  |  | 15571 | -0.664 | -0.6535 | No |
| 38 | TOM1L1 | na |  |  | 15829 | -0.710 | -0.6597 | No |
| 39 | ATP1A1 | na |  |  | 15995 | -0.743 | -0.6607 | Yes |
| 40 | SEC31A | na |  |  | 16038 | -0.756 | -0.6552 | Yes |
| 41 | TMX1 | na |  |  | 16097 | -0.769 | -0.6503 | Yes |
| 42 | KRT18 | na |  |  | 16203 | -0.788 | -0.6478 | Yes |
| 43 | SCRN1 | na |  |  | 16392 | -0.830 | -0.6491 | Yes |
| 44 | ARF1 | na |  |  | 16653 | -0.887 | -0.6537 | Yes |
| 45 | GLA | na |  |  | 16858 | -0.944 | -0.6547 | Yes |
| 46 | MAPK1 | na |  |  | 16876 | -0.948 | -0.6459 | Yes |
| 47 | SEC22B | na |  |  | 16882 | -0.950 | -0.6364 | Yes |
| 48 | TSPAN8 | na |  |  | 16912 | -0.960 | -0.6281 | Yes |
| 49 | AP1G1 | na |  |  | 17027 | -0.995 | -0.6239 | Yes |
| 50 | ARFIP1 | na |  |  | 17070 | -1.007 | -0.6158 | Yes |
| 51 | RAB14 | na |  |  | 17105 | -1.014 | -0.6072 | Yes |
| 52 | GALC | na |  |  | 17235 | -1.060 | -0.6031 | Yes |
| 53 | ATP7A | na |  |  | 17248 | -1.066 | -0.5928 | Yes |
| 54 | PAM | na |  |  | 17315 | -1.089 | -0.5851 | Yes |
| 55 | VPS4B | na |  |  | 17352 | -1.102 | -0.5758 | Yes |
| 56 | DNM1L | na |  |  | 17464 | -1.139 | -0.5699 | Yes |
| 57 | CLTC | na |  |  | 17555 | -1.181 | -0.5626 | Yes |
| 58 | M6PR | na |  |  | 17650 | -1.221 | -0.5550 | Yes |
| 59 | TPD52 | na |  |  | 17682 | -1.234 | -0.5440 | Yes |
| 60 | CLCN3 | na |  |  | 17722 | -1.250 | -0.5332 | Yes |
| 61 | SNAP23 | na |  |  | 17800 | -1.287 | -0.5241 | Yes |
| 62 | VAMP3 | na |  |  | 17806 | -1.290 | -0.5112 | Yes |
| 63 | STX12 | na |  |  | 17816 | -1.292 | -0.4984 | Yes |
| 64 | CD63 | na |  |  | 17821 | -1.295 | -0.4854 | Yes |
| 65 | CTSC | na |  |  | 17837 | -1.302 | -0.4728 | Yes |
| 66 | ATP6V1H | na |  |  | 18048 | -1.414 | -0.4694 | Yes |
| 67 | RAB2A | na |  |  | 18049 | -1.414 | -0.4549 | Yes |
| 68 | ZW10 | na |  |  | 18065 | -1.424 | -0.4411 | Yes |
| 69 | ARCN1 | na |  |  | 18180 | -1.486 | -0.4319 | Yes |
| 70 | AP2B1 | na |  |  | 18181 | -1.487 | -0.4167 | Yes |
| 71 | ARFGAP3 | na |  |  | 18214 | -1.508 | -0.4029 | Yes |
| 72 | ADAM10 | na |  |  | 18289 | -1.557 | -0.3909 | Yes |
| 73 | VAMP7 | na |  |  | 18298 | -1.562 | -0.3753 | Yes |
| 74 | STAM | na |  |  | 18314 | -1.580 | -0.3599 | Yes |
| 75 | NAPG | na |  |  | 18370 | -1.619 | -0.3462 | Yes |
| 76 | RPS6KA3 | na |  |  | 18422 | -1.654 | -0.3320 | Yes |
| 77 | COPB2 | na |  |  | 18424 | -1.654 | -0.3151 | Yes |
| 78 | USO1 | na |  |  | 18436 | -1.663 | -0.2987 | Yes |
| 79 | TMED10 | na |  |  | 18458 | -1.680 | -0.2826 | Yes |
| 80 | BNIP3 | na |  |  | 18492 | -1.701 | -0.2669 | Yes |
| 81 | PPT1 | na |  |  | 18555 | -1.749 | -0.2523 | Yes |
| 82 | LMAN1 | na |  |  | 18569 | -1.757 | -0.2350 | Yes |
| 83 | RAB5A | na |  |  | 18593 | -1.774 | -0.2180 | Yes |
| 84 | RAB22A | na |  |  | 18619 | -1.804 | -0.2009 | Yes |
| 85 | COPB1 | na |  |  | 18629 | -1.821 | -0.1827 | Yes |
| 86 | TMED2 | na |  |  | 18685 | -1.879 | -0.1664 | Yes |
| 87 | SOD1 | na |  |  | 18799 | -2.015 | -0.1517 | Yes |
| 88 | TSG101 | na |  |  | 18862 | -2.104 | -0.1334 | Yes |
| 89 | YIPF6 | na |  |  | 18895 | -2.160 | -0.1130 | Yes |
| 90 | SNX2 | na |  |  | 18901 | -2.170 | -0.0910 | Yes |
| 91 | SCAMP1 | na |  |  | 19017 | -2.402 | -0.0725 | Yes |
| 92 | LAMP2 | na |  |  | 19026 | -2.430 | -0.0480 | Yes |
| 93 | BET1 | na |  |  | 19035 | -2.469 | -0.0232 | Yes |
| 94 | AP3S1 | na |  |  | 19148 | -3.097 | 0.0026 | Yes |
Table: GSEA details [plain text format]

  

Fig 2: HALLMARK\_PROTEIN\_SECRETION      
 Blue-Pink O' Gram in the Space of the Analyzed GeneSet

  

Fig 3: HALLMARK\_PROTEIN\_SECRETION: Random ES distribution      
 Gene set null distribution of ES for **HALLMARK\_PROTEIN\_SECRETION**
